# Molecular strategies of *Salmonella* Typhimurium: from host cell invasion and metabolic hijacking to immune evasion

**DOI:** 10.3389/fcimb.2026.1753120

**Published:** 2026-03-27

**Authors:** Meihong Fu, Yuling Sun, Yingquan Wu, Nanlong Zhou, Meiling Huang, Tiansen Li

**Affiliations:** 1 School of Tropical Agriculture and Forestry, Hainan University, Haikou, China; 2 Department of Basic Medicine, Hainan Vocational University of Science and Technology, Haikou, China; 3 School of Pharmacy, Hainan Medical University, Haikou, China

**Keywords:** effector proteins, immune evasion, intracellular survival, metabolic reprogramming, *Salmonella* Typhimurium, T3SS

## Abstract

*Salmonella enterica* serovar Typhimurium (*S*. Typhimurium), a prevalent zoonotic pathogenic bacterium, has its pathogenicity and intracellular parasitic mechanisms intricately linked to a range of virulence factors. *S*. Typhimurium infects the host mainly through the type III secretion system (T3SS), with SPI-1-encoded T3SS mediating bacterial internalization and host cytoskeletal remodeling, while SPI-2-encoded T3SS maintains the stability of bacterial-containing vesicles (SCVs) by inhibiting lysosomal fusion. Moreover, the type VI secretion system (T6SS) aids *S*. Typhimurium in competing with other microorganisms and facilitates its ability to traverse the mucus layer for host colonization, with flagella also contributing significantly to this process. Various effector proteins, secreted by *S*. Typhimurium, help preserve membrane stability for intracellular survival. To sustain intracellular viability, *S*. Typhimurium stabilizes its membranes by releasing multiple effector proteins. It sustains the homeostasis of its intracellular living environment by hijacking the host’s glycolytic metabolism and suppressing immune responses. At the same time, *S*. Typhimurium also uses the bidirectional regulation of the autophagy pathway and the co-regulation of apoptosis and pyroptosis programs to construct complex immune escape routes. Our primary objective is to integrate cutting-edge research on *S*. Typhimurium’s parasitism within host cells and the bidirectional mechanisms governing its coexistence with the host. Our focus lies on secretion systems, metabolic reprogramming, and immune evasion, which could provide new perspectives for developing approaches to combat and manage *S*. Typhimurium.

## 1 Introduction

Transmitted primarily via contaminated food or water, the intracellular pathogen *S*. Typhimurium (a member of non-typhoidal *Salmonella*, NTS) causes gastrointestinal and systemic illnesses in humans and animals, including diarrhea, abdominal pain, and potentially sepsis. Data reveal some a global total of 93.8 million cases of NTS gastroenteritis, among which 80.3% stem from foodborne infections. This translates to roughly 155,000 yearly fatalities, imposing a substantial disease burden globally across developed and developing nations (Majowicz et al., 2010). According to statistics from relevant authorities, there are approximately 135 cases of NTS in the United States each year (Collier et al., 2021); In sub-Saharan Africa, the mortality rate of *Salmonella* infections reaches as high as 15% - 20% (Crump et al., 2023). In China, Chen et al. found in a statistical study that the probability of children being infected with NTS has increased significantly (Chen et al., 2023); Similarly, clinical studies have further documented a growing trend of multidrug resistance in isolated *S*. Typhimurium strains (Zhou et al., 2024; Gao et al., 2025). *Salmonella*’s high infectivity stems from its sophisticated pathogenic strategies, which enable it to colonize and thrive within host environments. Initially, the bacterium attaches to intestinal lining cells via surface structures like fimbriae and outer membrane proteins, facilitating close contact with the host. Subsequently, the bacterium employs the T3SS-1 to transport effector proteins into host cells. These effectors induce actin filament rearrangements and membrane ruffling, triggering bacterial engulfment and formation of SCVs. Usually, *Salmonella* evades host immune clearance by entering these special compartments. However, once *Salmonella* successfully enters host cells, it utilizes effector proteins secreted from T3SS-2 to regulate the state of the SCVs, mainly for the further reproduction of the bacterium. These dynamically regulated vacuoles create highly secure microenvironmental conditions for the bacteria to survive within the cells (Hueck, 1998; Lou et al., 2019); *S*. Typhimurium achieves intracellular persistence by reorganizing the host’s organelles via its SCVs. This compartment serves as a protected replicative hub where the pathogen orchestrates metabolic adaptation and immune evasion. To subvert host clearance mechanisms, *Salmonella* delivers effector proteins via T3SS, enabling escape from innate immune surveillance Contemporary research has evolved beyond singular virulence factor analysis, integrating multifactorial investigations of bacterial secretion systems, host metabolic reprogramming, and SCVs-centered immune evasion networks.

Therefore, this review first establishes a conceptual framework for the stage-specific virulence programs employed by *S*. Typhimurium. Subsequently, it will systematically elaborate on how it achieves intracellular parasitism using secretion systems such as T3SS, with a focus on the maintenance of SCVs, the reprogramming of host metabolism, and the bidirectional regulation of cell death pathways such as autophagy, apoptosis, and pyroptosis. It aims to provide a relevant theoretical basis for the development of new anti-infective strategies.

## 2 Conceptual framework: stage-specific virulence programs and minimal virulence networks

**Figure 1:**
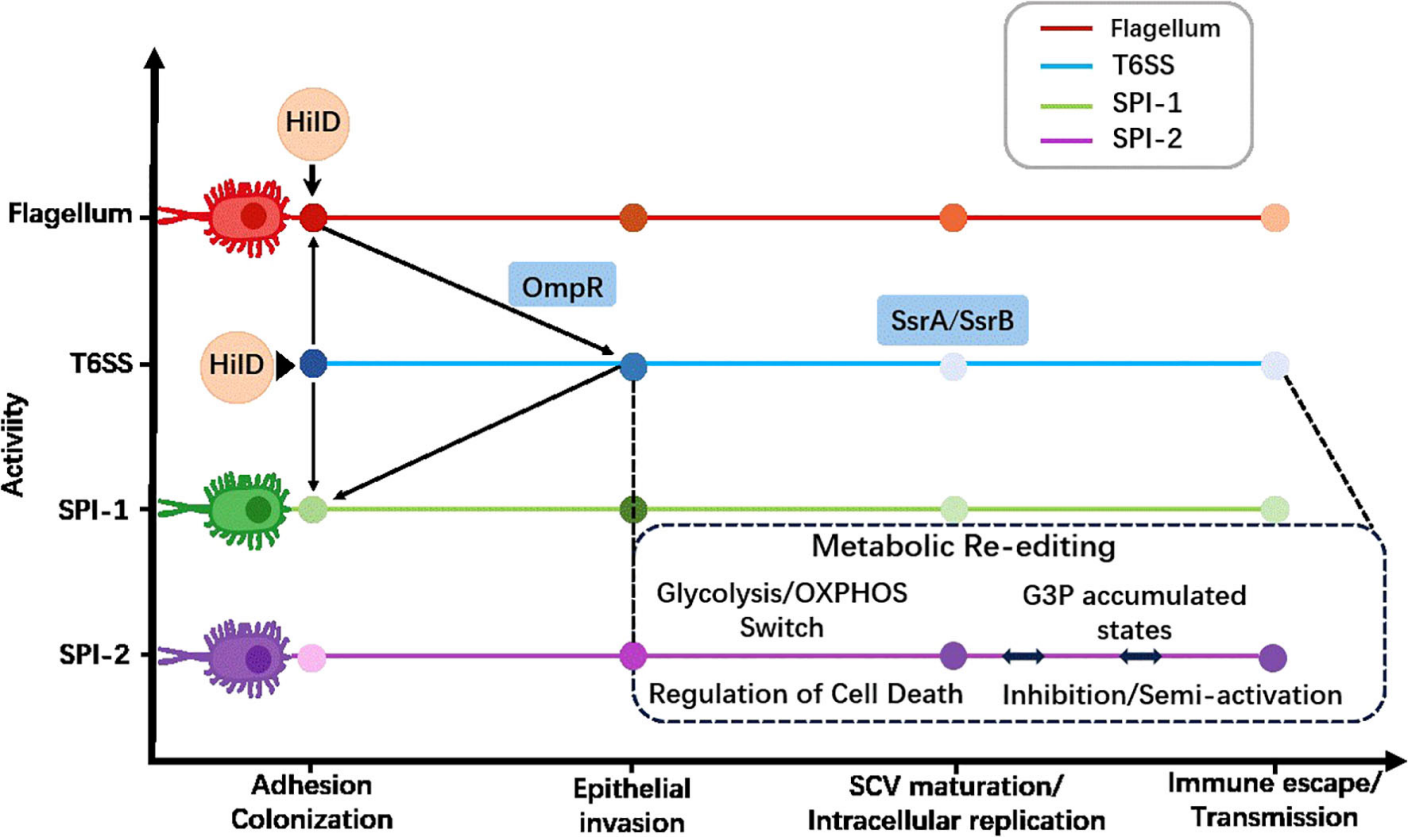
Stage-specific virulence program of *S*. Typhimurium. This figure shows the temporal coordination of major virulence systems during key stages of infection. The relative activity of each system is represented by different colors. Arrows indicate key regulatory transition nodes. The sequential deployment of these specialized modules (flagella/T6SS for colonization, SPI-1 for invasion, and SPI-2 for intracellular survival) embodies a virulence program that optimizes resource utilization and host adaptation. The shade of color indicates the activity level of the system.

The successful colonization of *S*. Typhimurium does not rely on the simultaneous expression of all virulence factors, but rather on a complex stage-specific virulence program (**Figure 1**). This program can be conceptualized as a minimal virulence network, where core regulatory hubs (such as HilD and SsrB) integrate host microenvironmental signals to sequentially deploy different virulence modules in a spatiotemporal manner. This dynamic, hierarchical control ensures efficient resource allocation and optimal adaptation throughout the infection cycle. The infection process can be divided into four interrelated stages, each with unique molecular strategies and key virulence systems.

### 2.1 Colonization and invasion

The primary goal of this initial stage is to breach host barriers, adhere to intestinal epithelial cells, and trigger active internalization. The flagellar system facilitates bacterial motility and mucus penetration. T6SS provides a competitive advantage by antagonizing the commensal microbiota, aiding in niche establishment. Subsequently, T3SS encoded by SPI-1 becomes the core executor. It remodels the host’s actin cytoskeleton through the injection of relevant effector proteins, ultimately forming the protective SCVs.

### 2.2 Intracellular adaptation and SCV maturation

Upon entering the host cell, the bacterial program rapidly shifts from invasion to intracellular persistence. The T3SS encoded by SPI-2 becomes the primary virulence apparatus. Its effector proteins actively remodel the nascent SCV, anchoring it to the host’s Golgi apparatus, inhibiting lysosomal fusion, and creating a specialized, replicative niche that protects the bacteria from host defense mechanisms.

### 2.3 Metabolic hijacking and immune regulation

In order to obtain the energy required for replication within the safe SCV, *S*. Typhimurium actively reprograms the metabolism of host cells. Some effector proteins manipulate host signal transduction to induce a shift in glycolysis, thereby redirecting metabolites for bacterial catabolism. Meanwhile, this pathogen employs a multi-faceted strategy to create an immune environment conducive to its own survival. This includes bidirectional regulation of autophagy (inhibiting its bactericidal function while potentially utilizing its components) and regulation of programmed cell death pathways (apoptosis, pyroptosis, necroptosis) to suppress inflammation, delay cell death, and acquire resources.

### 2.4 Dissemination and systemic immune evasion

To promote dissemination within the host, *S*. Typhimurium utilizes late-stage effector proteins to regulate host cell fate and systemic immunity. Some effector proteins can trigger pro-inflammatory cell death modalities (such as necroptosis), causing lysis of exhausted host cells and facilitating bacterial release. Additionally, certain effector proteins can directly impair adaptive immunity by downregulating MHC class II molecule-mediated antigen presentation, enabling the bacteria to persist systemically.

This pathogenic framework, which is executed in stages and by modules, indicates that *Salmonella* infection does not follow simple linear steps, but rather resembles a network where various parts are closely linked and dynamically adjusted. The following text will deeply analyze the specific roles of each virulence system and how they synergistically integrate, clarifying how bacteria can successfully achieve infection with limited virulence components through precise timing control.

## 3 Dual function of The T3SS

### 3.1 SPI-1 type 3 secretion system: initial invasion

**Figure 2:**
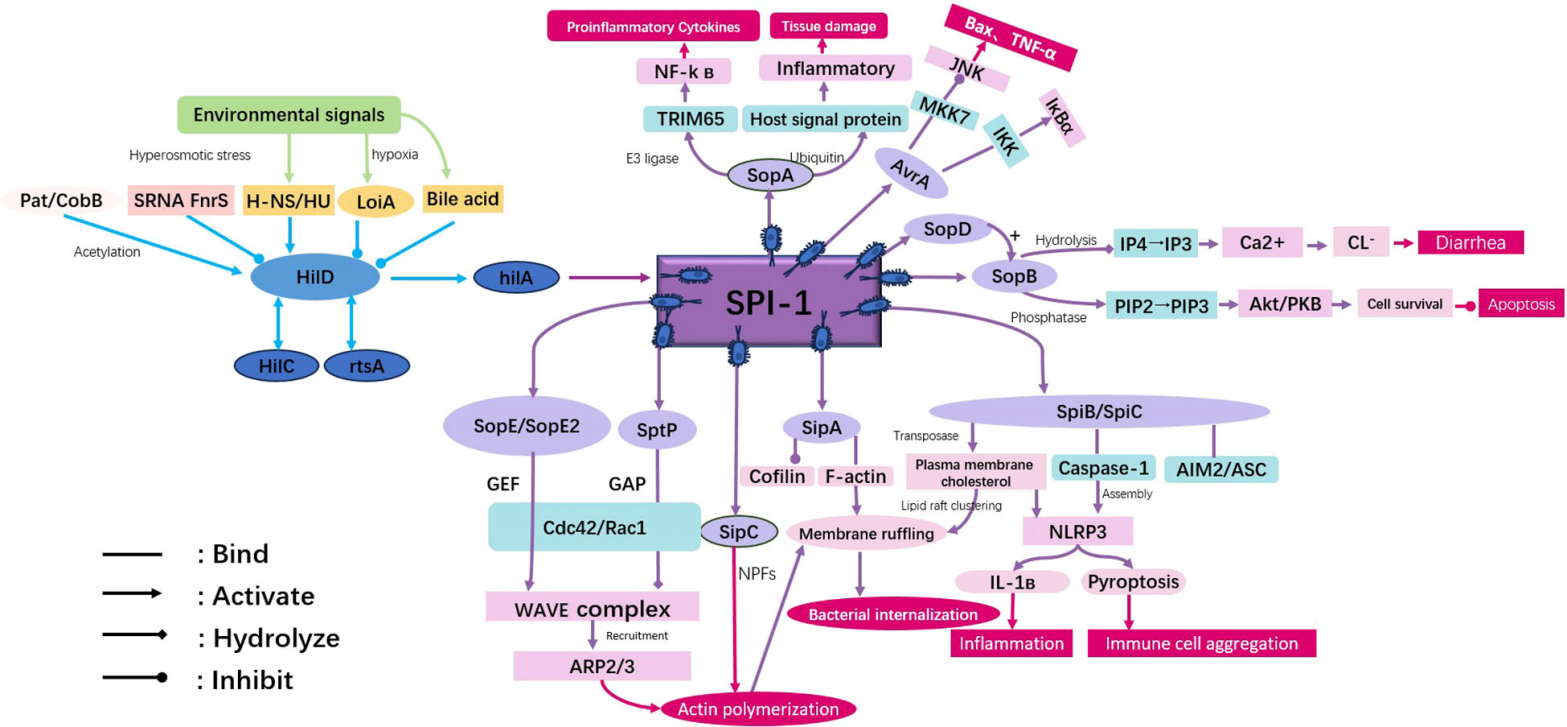
Effector proteins of SPI-1 and their action mechanisms. The infection process of *Salmonella* SPI-1 T3SS is regulated by the Hil cascade, and its secretion apparatus injects multiple effector proteins into host cells to form a synergistic effect.

The SPI-1-encoded T3SS plays a crucial role in mediating the early invasion of host cells by bacteria through the secretion of effector proteins (**Figure 2**). The SPI-1 fragment is approximately 40 kb in length and contains 39 genes. These genes not only encode operons related to the T3SS assembly, such as prg, inv, and sic, but also encode a series of effector proteins including SipA, SipC, SopE, SopE2, SptP, and AvrA. These effector proteins work synergistically to drive actin polymerization, activate Rac1/Cdc42 signaling, and reshape the cytoskeleton, providing key support for rapid bacterial invasion and SCVs formation (Lerminiaux et al., 2020).

To achieve this key invasion process, it relies on the transcriptional network regulated by SPI-1. Among them, the AraC-type regulatory factor HilD is the most upstream activator of this cascade. It can directly or indirectly activate the expression of the hilC, rtsA, and hilA genes by antagonizing the inhibitory effect of H-NS, and it is also a key point for most environmental signals. HilC and RtsA form a positive feedback loop with HilD, which amplifies the HilD signal while achieving self-activation and mutual activation, and both can form homodimers or heterodimers with HilD (Kalafatis and Slauch, 2021). The main transcriptional activator HilA is regulated by the three-level signal amplification of HilD-HilC-RtsA. On the one hand, it directly drives the expression of T3SS structural genes such as prg/org, inv/spa, and sic/sip; on the other hand, it indirectly regulates effector protein genes through the AraC/XylS family regulatory factor InvF. InvF needs to first form a complex with the chaperone protein SicA to specifically activate effector protein genes such as sip and sop. This constitutes a typical feed-forward loop of “HilD→HilC/RtsA→HilA→T3SS structural and effector genes” (Kalafatis and Slauch, 2021; Liang et al., 2023). At the same time, the activity of HilD is regulated at multiple levels, including transcription, post-translational modification, and post-transcriptional regulation mediated by small RNAs (sRNAs).

Building on the regulation at the transcriptional level, sRNA-mediated post-transcriptional regulation further enhances the dynamic flexibility of SPI-1 activation. Spot 42 and SdsR can bind to the 3′ UTR region of *hilD* mRNA through Hfq-mediated base pairing, masking the cleavage site of RNase E to improve the stability of *hilD*, thereby promoting SPI-1 expression (Abdulla et al., 2023). A recent study found that InvR can directly interact with *hilA* mRNA and refine the expression level of HilA through negative feedback regulation (Hou et al., 2025). Another study showed that S1K247 can regulate the translation efficiency of *hilD* through reversible lysine acetylation, thereby affecting the activation of SPI-1 (Joiner et al., 2023). These sRNAs help bacteria quickly switch virulence states in different host microenvironments by regulating the mRNA stability of *hilD* or *hilA*.

In addition to regulation at the mRNA level, post-translational modification (PTM) of proteins also helps HilD achieve rapid activity switching under different metabolic states at the protein activity level, thereby balancing bacterial virulence and growth. Pat can mediate lysine acetylation of HilD and S1K247. This modification can not only enhance the stability of HilD but also regulate the function of ribosomal protein S1, thereby affecting flagellar assembly and arginine biosynthesis. Moreover, this acetylation process is independent of CobB deacetylation, forming an independent regulatory pathway (Hung et al., 2016; Sang et al., 2016). Propionyl-CoA reduces the stability of HilD through propionylation, exerting a negative regulatory effect. AvrA inhibits JNK phosphorylation through its acetyltransferase activity, indirectly maintaining the homeostasis of the actin cytoskeleton (Lin et al., 2016).

As the core hub of the SPI-1 regulatory network, HilD can also integrate signals from various environmental factors to form an integrated regulatory network from “environmental sensing to transcriptional activation”, so as to adapt SPI-1 expression to different environmental conditions. Under hypoxic/low-oxygen conditions, the ArcB/ArcA two-component system (TCS) activates the *loiA* gene to indirectly enhance *hilD* expression, while inhibiting the Lon protease to increase the level of HilD protein; bile acids can promote the expression of virulence genes by weakening the inhibitory effect of HilD on SPI-1 genes; under conditions of osmotic pressure changes or in AT-rich regions, H-NS forms an inhibitory complex on the promoters of *hilD*, *hilC*, and *hilA*, and HilD relieves this inhibition by antagonizing H-NS (Liang et al., 2023); the diffusible signal factor (DSF) can directly bind to HilD to regulate its activity, thereby determining the timing of bacterial invasion (Saleh et al., 2023); long-chain fatty acids (LCFAs) inhibit the functions of HilD and HilC, reducing the expression of SPI-1 (Joiner et al., 2023).

Under the synergistic action of the multi-level regulatory network involving the aforementioned transcription, post-transcription, post-translational modifications, and integration of environmental signals, the expression of various effector proteins is regulated, and through division of labor and collaboration, they promote bacteria to complete the invasion process. SipA binds to actin monomers to enhance fiber stability, while SipC directly interacts with actin filaments to promote the formation of filament bundles. At specific concentrations, the two synergistically enhance actin polymerization and inhibit the depolymerization activity of ADF/cofilin (Srikanth et al., 2010; Niedzialkowska et al., 2024). As guanine nucleotide exchange factors (GEFs), SopE and SopE2 activate Rac1 and Cdc42 respectively, induce the assembly of the N-WASP-Arp2/3 complex, and drive the rapid polymerization of new actin filaments (Williams et al., 2004; Zhao et al., 2025). SptP inactivates Rac1/Cdc42 through its GTPase-activating protein (GAP) activity, which not only reduces actin assembly but also downregulates the JNK/p38 signaling pathway, helping to restore host cell homeostasis after bacterial invasion (Tang et al., 2015).

### 3.2 SPI-2 type 3 secretion system: intracellular maintenance

The pathogenicity of *S*. Typhimurium depends on multiple pathogenicity islands on its chromosome. Among them, the T3SS encoded by SPI-2 is a key molecular device for the long-term survival, reproduction, and systemic spread of the bacteria within host macrophages. This system primarily fulfills its core functions by regulating the stability of the SCVs and evading host immune mechanisms (Buckner et al., 2011). The functional core of SPI-2 relies on the SsrA/SsrB two-component regulatory system. SsrA can sense environmental changes in host cells, such as low Mg^2+^ and acidic pH. After phosphorylation, it activates SsrB, which in turn relieves the inhibitory effect of H-NS on SPI-2 gene transcription and recruits RNA polymerase to initiate the expression of T3SS-related genes (Garmendia et al., 2003; Valvano, 2022). It is worth noting that HilD, traditionally considered the main regulator of SPI-1, also plays an important role in SPI-2 regulation (He et al., 2025). Studies have shown that HilD can not only form a complex with OmpR to synergistically promote the transcriptional switch from SPI-1 to SPI-2 in *Salmonella* but also may directly bind to the *ssrA/B* promoter region to enhance the transcriptional activity of SsrB (Lee et al., 2000; Garmendia et al., 2003). However, the experimental evidence for the direct binding of HilD to the *ssrA/B* promoter is insufficient, and this mechanism still needs to be further confirmed through high-resolution experiments. In addition to core regulatory factors, SPI-2 is subject to sophisticated multi-level regulation. The PhoP/PhoQ two-component system can indirectly regulate SPI-2 by upregulating the expression of ssrA/B; recent studies have shown that the quorum sensing signal molecule AI-2 can regulate the transcription of ssrA/B through PhoP; small RNAs such as ArcZ, FnrS, SdsR, and Spot42 indirectly affect the activation of SPI-2 by regulating *hilD* translation; ppGpp/DksA can strictly respond to nutritional stress and promote SsrB-mediated transcription initiation (Valvano, 2022; He et al., 2025); meanwhile, oxidative stress signals such as high concentrations of H_2_O_2_ can inhibit the expression of SPI-2 genes, suggesting a negative correlation between reactive oxygen species and SPI-2 regulation (Chen et al., 2025). It should be emphasized that the above regulatory network shows certain variations among different *Salmonella* strains and different host cell types, and thus needs to be interpreted in combination with the actual situation in specific experimental systems.

SPI-2 effector proteins assist *Salmonella* in stably surviving within host cells through various molecular strategies. SseF, SseG, and SopD2 maintain the structural integrity of the SCVs by regulating vesicle trafficking and anchoring to the Golgi apparatus; the deletion of these genes leads to a significant reduction in the replication ability of bacteria within macrophages (Pillay et al., 2023). SteD inhibits the expression of host MHC class II molecules and blocks the antigen presentation process, while SpvB, an ADP-ribosyltransferase, modifies Arg177 of host G-actin, disrupting actin polymerization. This cytoskeletal disruption not only aids in immune evasion and suppresses inflammatory signals but also has downstream consequences for autophagy and host cell death pathways (Ma S. et al., 2025). SseK1 interferes with the NF-κB and MAPK pathways through arginine GlcNAcylation of host signaling molecules to weaken inflammatory responses (Jennings et al., 2017). Recent studies have also revealed the important role of metabolic reprogramming in SPI-2 function. *Salmonella* can utilize β-alanine produced by the host and itself to promote its replication within macrophages, and can also hijack host GLUT1/GLUT3 transporters to obtain glucose. These metabolic adaptation strategies further enhance the biological functions of SPI-2 effector proteins (Meng et al., 2023). From an evolutionary perspective, the excellent environmental adaptability and intracellular survival ability of *S*. Typhimurium mainly depend on the conserved gene fragments in SPI-2. Multiple studies have confirmed that SPI-2 mutant *S*. Typhimurium have significantly impaired survival ability in macrophages, which genetically verifies the core role of SPI-2 in systemic *Salmonella* infections (Hensel et al., 1999; Noster et al., 2019a).

## 4 Auxiliary virulence function of T6SS

In addition to the T3SS, which plays a central role in invasion and intracellular survival, S. Typhimurium also utilizes T6SS, a contact-dependent weapon, to expand its ecological niche and consolidate infections. Unlike the T3SS, which primarily targets host cells, the T6SS is more involved in interbacterial competition and provides strong support for the initial colonization and subsequent systemic infection of bacteria by affecting the host mucosal barrier (Wang et al., 2011; Wei et al., 2020). T6SS of *S*. Typhimurium is a nano-injection device assembled from at least 13 conserved core proteins. Among them, Hcp forms a hollow tubular structure, and VgrG constitutes a perforating tip cap (Wang Z. et al., 2020). Gene knockout experiments showed that the deletion of hcp or vgrg significantly reduced the ability of *Salmonella* to adhere to and invade HeLa epithelial cells, and the survival rate in raw 264.7 macrophages also decreased significantly, indicating that these two core proteins play a key role in the processes of adhesion, invasion, and anti-phagocytosis (Wang P. et al., 2020). Further transcriptional analysis found that the deletion of *hcp1* led to downregulated expression of *rpoS*, the adhesion factor *fimH*, and multiple flagellar genes, thereby affecting bacterial motility and adaptability to the host. Additionally, there is mutual regulation among members of the hcp family; the deletion of one hcp causes transcriptional compensation of other hcp genes, suggesting that T6SS achieves functional diversification through various Hcp combinations (Flaugnatti et al., 2020). Structural studies revealed that the unique VgrG (VgrS) of *Salmonella* has an open gp27 head domain and a unique C-terminal tail, which can specifically load a variety of effectors and undergo open-closed conformational transitions during assembly. This provides a molecular basis for the effector loading mechanism (Chen et al., 2023).

At the regulatory level, the iron ion-responsive system Fur directly binds to the *clpV* promoter and inhibits its transcription when iron is sufficient. Iron deficiency or inactivation of Fur function relieves this inhibition, promoting T6SS assembly and enhancing interbacterial competition and pathogenicity (Wang et al., 2007; Wang et al., 2019). RcsB, as the core regulator of the multicomponent phosphotransduction system, mainly regulates colanic acid synthesis and flagellar genes, but in a highly activated state, it can also inhibit SPI-2 and indirectly affect T6SS expression in SPI-6, forming a negative regulatory network for T6SS activity (Journet and Cascales, 2016). In addition, H-NS limits the transcription of T6SS genes through its silencing effect in the SPI-6 region, and cyclic di-GMP can relieve the inhibition of H-NS, thereby restoring T6SS function (Barbosa and Lery, 2019). Although PmrA mainly regulates lipopolysaccharide modification and the SPI-1/2 T3SS, its regulation of LPS indirectly affects bacterial surface properties, which may in turn produce synergistic or antagonistic effects on T6SS activation (Choi and Groisman, 2013).

## 5 The critical role of flagella

During the colonization of *S*. Typhimurium in the host, its flagella, as complex nanomachines, play an indispensable role. This organelle consists of three core components: the flagellar filament, the flagellar hook, and the basal body. Among them, the helical flagellar filament is mainly composed of two flagellin subunits, FliC and FljB. The methylation of lysine residues mediated by FliB can significantly enhance its surface hydrophobicity, thereby strengthening the adhesion and invasion of bacteria to host epithelial cells (Horstmann et al., 2020; Yamaguchi et al., 2020). The middle hook part is composed of FlgE protein, which is responsible for connecting the filament to the basal body and exhibits adjustability in length and assembly under temperature and acidic conditions. The basal body contains a motor that drives the rotation of the flagella (helping *Salmonella* reach intestinal epithelial cells) and a dedicated type III secretion system (fT3SS), which can secrete effector proteins when invading the host (Uchida et al., 2013; Minamino and Imada, 2015). The core regulatory factor for flagellar gene expression is the FlhD_4_C_2_ complex, which initiates the synthesis of various flagellar structures by activating class II genes (Tomoyasu et al., 2003). Its expression is subject to multiple layers of regulation. For example, the positive regulatory factor AsiR can directly bind to the *flhDC* promoter to promote flagellar gene transcription. However, in the acidic intracellular environment of macrophages (pH≈5), the AsiR gene is inhibited, leading to decreased expression of *flhDC* and its downstream genes through an as-yet unclear mechanism, which reduces the release of pro-inflammatory cytokines and helps bacteria evade immune surveillance (Ma et al., 2021).

**Figure 3:**
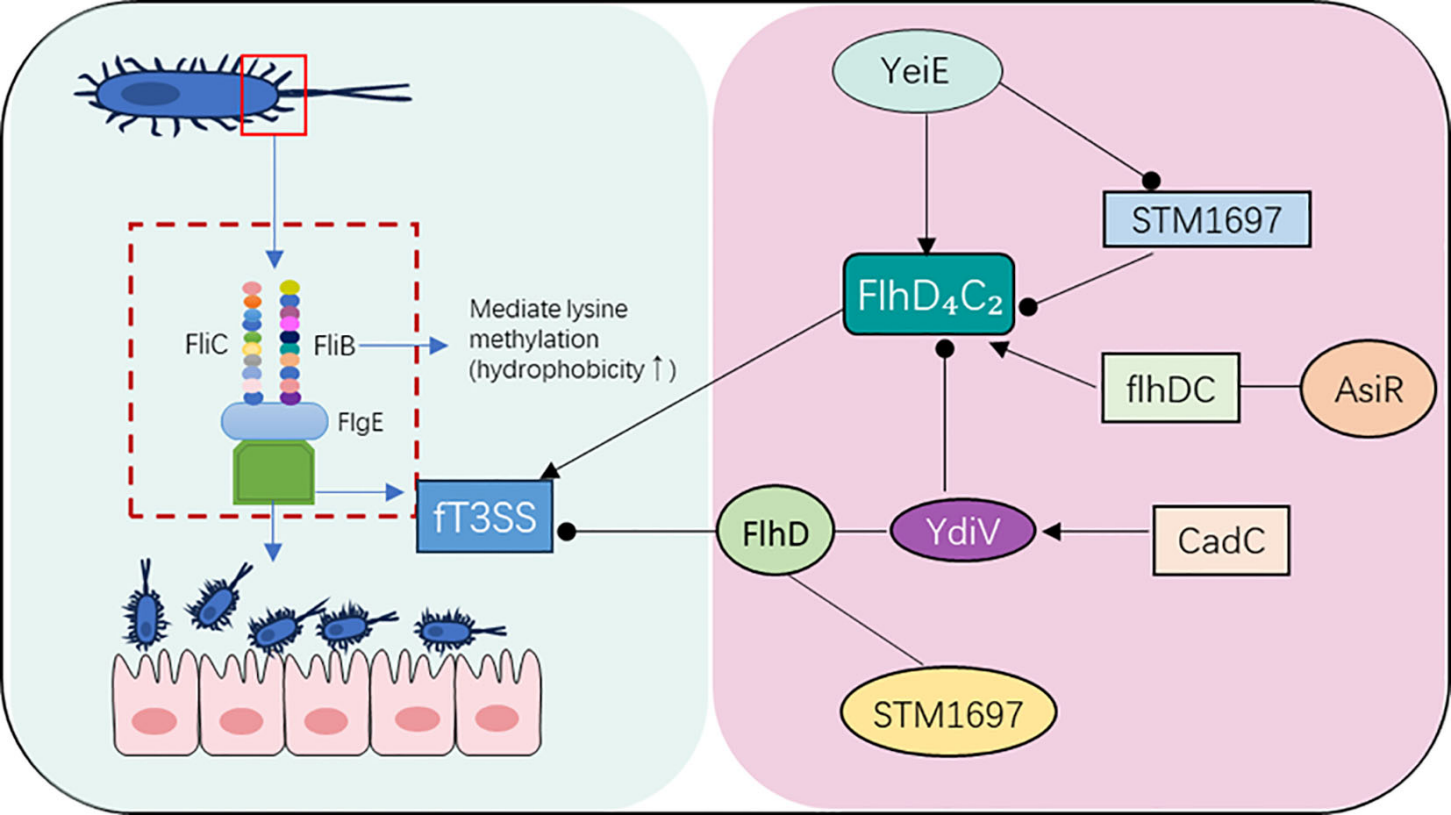
Flagellar structure of *S*. Typhimurium and its association with motility regulation. Left: Three-dimensional structure of the flagellum—including the FliC/FljB-polymerized filament (FliB-mediated lysine methylation enhances hydrophobicity), FlgE-composed hook (connecting filament and basal body), and basal body (containing the rotation motor and fT3SS). Right: Regulatory network centered on the FlhD_4_C_2_ complex: YeiE relieves STM1697-mediated inhibition of FlhD_4_C_2_; AsiR positively regulates FlhD_4_C_2_ (inactivated under acidic conditions); CadC-mediated YdiV and STM1697 directly inhibit FlhD_4_C_2_ function.

Meanwhile, acid signals further inhibit flagellar synthesis through the CadC-YdiV axis. Specifically, acidic conditions activate CadC, which then upregulates the transcription of YdiV. The YdiV protein binds to FlhD_4_C_2_ via its EAL domain and prevents it from recruiting RNA polymerase, thereby achieving the shutdown of flagella under nutrient and acid stress (Wada et al., 2011; Wang et al., 2022). In addition, YdiU can UMPylate the Ser31 site of the FlhC subunit, directly disrupting the binding of FlhDC to target promoters and providing a post-translational shutdown pathway. STM1697 (another EAL-like protein) can also restrict the recruitment of RNA polymerase by FlhD_4_C_2_ through interaction with FlhD, inhibiting flagellar gene expression and reducing immune visibility (Li et al., 2017; Westerman et al., 2021). There is also cross-regulation between flagella and pathogenicity SPI-1. HilD, the master regulator of SPI-1, can activate *flhDC* transcription, but its activation leads to a decrease in SPI-1-dependent proton motive force and an upregulation of adhesion factors, resulting in motility defects in bacteria during the invasion stage while enhancing adhesion to epithelial cells. In the hypoxic intestinal environment, the global oxygen-sensing regulator FNR directly regulates *flhDC* and various virulence genes in SPI-1, further coupling flagellar synthesis with anaerobic metabolism and virulence (Li et al., 2018; Jiang et al., 2019). **Figure 3** briefly depicts the flagellar structure and its relationship with motility regulation. The flagellar system not only endows *Salmonella* with motility and initial adhesion capabilities, but its structure and regulation also have extensive cross-talk with the T3SS, jointly coordinating the infection process of bacteria from extracellular to intracellular. After successfully invading and colonizing host cells, establishing and maintaining a suitable intracellular survival microenvironment becomes the primary task.

## 6 Maintain the intracellular survival microenvironment

### 6.1 Dynamic regulatory network of Salmonella-containing vacuoles

After successfully invading host cells, the core way for *S*. Typhimurium to survive is to construct and maintain a special membrane structure, namely the SCV. The SCV is not a static organelle but a continuously changing and regulated microenvironment. It initially originates from the endocytic vesicle formed by the invagination of the cell membrane during bacterial invasion. Subsequently, under the synergistic effect of various bacterial effector proteins, the SCV can avoid fusion with lysosomes and gradually mature into a sanctuary that is conducive to bacterial reproduction and can resist host immune attacks. In some cases, some bacteria can also actively leave the SCV, enter the cytoplasm, and switch to another survival mode. Therefore, understanding the dynamic changes of the SCV is very important for revealing the intracellular survival mechanism of *Salmonella*. The following text will elaborate on the key regulatory events in this process in stages.

#### 6.1.1 Formation of early SCV

During the infection process of *S*. Typhimurium, the formation of SCVs is a crucial step for its intracellular survival. In the early stage of SCV formation, the effector protein SopB plays a central role as a phosphoinositide phosphatase. It initiates the signaling pathway for SCV formation by dephosphorylating PI (4,5) P_2_, triggering changes in membrane lipid composition, reducing membrane surface charge, and promoting the accumulation of PI (3) P. This process also promotes the recruitment of host sorting nexins SNX1 and SNX3 to the SCV membrane, forming a tubular membrane network, which enhances the membrane contraction and physical barrier function of SCVs (Knodler et al., 2009; Bakowski et al., 2010). In addition, SopB can specifically bind to the cytoskeletal protein vimentin, enhancing the structural integrity of SCVs and reducing the risk of their intracellular rupture (Stévenin et al., 2019). SopB also inhibits cell apoptosis by regulating host signaling pathways, such as interacting with CDC42 and activating Akt signaling, thereby providing a favorable intracellular environment for the establishment of SCVs (Braun et al., 2010).

#### 6.1.2 Maturity and stability of SCV

As SCVs gradually mature, *Salmonella* mobilizes a variety of effector proteins to act together to prevent their fusion with lysosomes, thereby maintaining the stability of SCVs. SifA binds to the host protein SKIP (PLEKHM2) and may regulate its interaction with Rab9 and M6PR through SUMOylation modification, thereby blocking the fusion of SCVs with lysosomes, maintaining the low-acid state of SCVs, and promoting the formation of Salmonella-induced filaments (SIFs) (Diacovich et al., 2009; Chandrasekhar et al., 2023; McGourty et al., 2012). SopD2, as a GTPase-activating protein (GAP) for Rab7 (and Rab32), interferes with the endosome-lysosome transport process mediated by RILP and FYCO1 by inhibiting the nucleotide exchange of Rab7, effectively preventing SCVs from being degraded by lysosomes (D’Costa et al., 2015; Spanò et al., 2016; Bayer-Santos et al., 2016). SseF and SseG anchor SCVs to the microtubule network around the Golgi apparatus through interaction with the Golgi-associated protein ACBD3, which not only maintains the integrity of the SCV membrane structure but also synergistically inhibits the initiation of autophagy mediated by Rab1A (Agbor and McCormick, 2011; Yu et al., 2016). The outer membrane protein OmpA inhibits the recruitment of LAMP-1 to the SCV membrane through its extracellular loop structure, reduces the infiltration of host defense factors, and forms a physical barrier to enhance the resistance of SCVs to autophagy (Roy Chowdhury et al., 2022).

In addition to protein effectors, non-coding RNAs also play a regulatory role in the maturation process of SCVs. STnc3020 is highly expressed in the SPI-1 region and can directly bind to the 5’-UTR region of *prgJ* mRNA, regulating the translation efficiency of the T3SS rod protein PrgJ, thereby affecting the assembly of the T3SS apparatus, the secretion of effector proteins, and the maturation process of SCVs (Ma Z. et al., 2025).

#### 6.1.3 Cytoplasmic escape and immune evasion

For *Salmonella* whose SCVs have escaped into the cytoplasm, the secreted effector proteins help achieve immune evasion. As described in Section 4, the ADP-ribosyltransferase SpvB inhibits actin polymerization, thereby blocking the formation of autophagosomes and enhancing the survival ability of bacteria in the cytoplasm (Barth and Aktories, 2011; Chu et al., 2016). In addition, the effector protein SteD secreted by SPI-2 hijacks the host E3 ubiquitin ligase MARCH8 through its transmembrane domain, leading to the ubiquitination and degradation of MHC class II molecules and co-stimulatory molecule B7-2, impairing the antigen presentation and immunological synapse formation of dendritic cells, inhibiting T cell activation, and constructing a systemic immune evasion network (Bayer-Santos et al., 2016).

### 6.2 Metabolic reprogramming strategies

**Figure 4:**
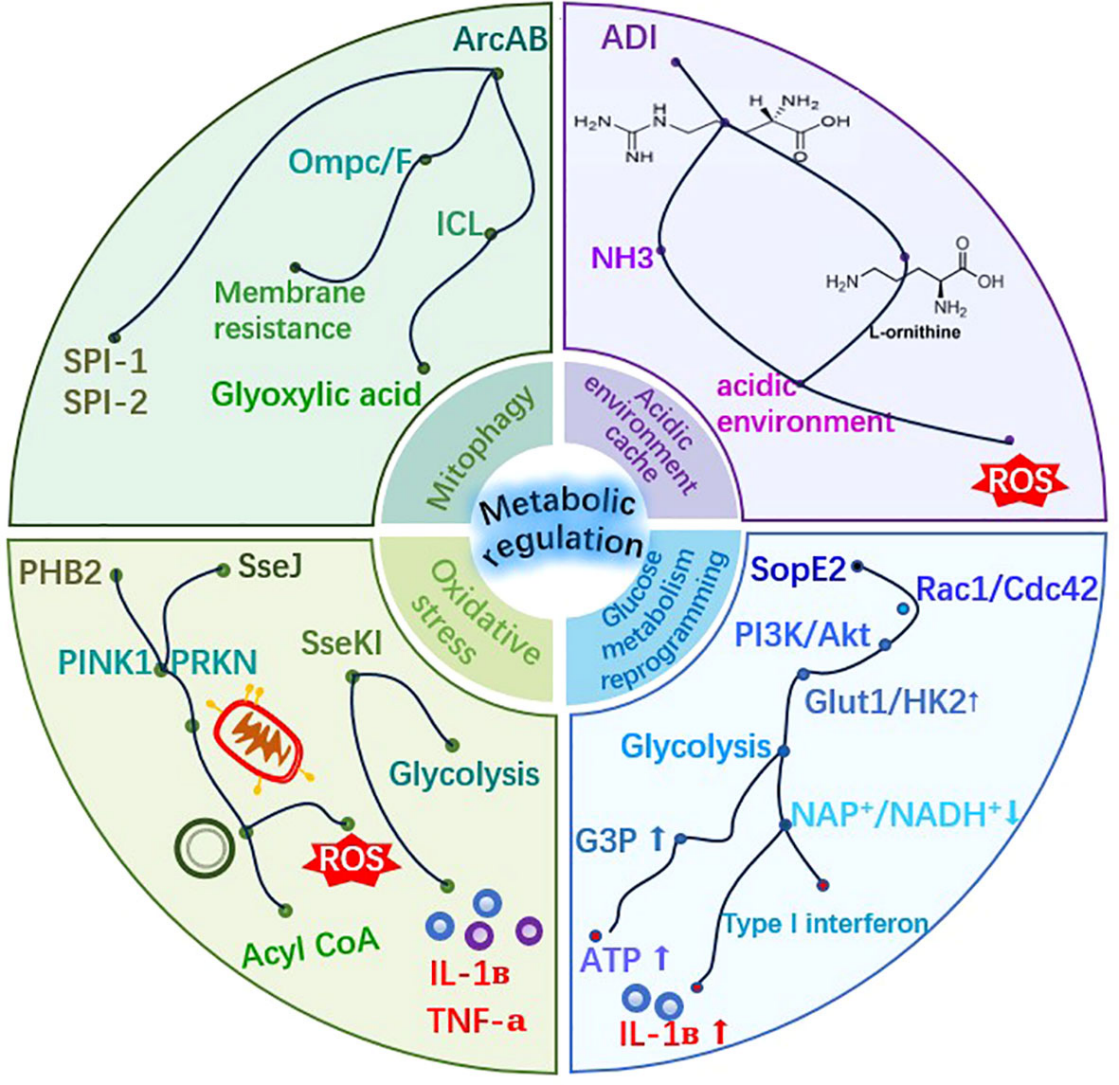
Metabolic reprogramming strategy diagram of *S*. Typhimurium. *S*. Typhimurium can utilize multiple metabolic pathways to provide a favorable environment for its survival in host cells.

It should be noted that specific metabolic dependencies and reprogramming strategies of *S*. Typhimurium may vary among different strains (e.g., SL1344 vs. ATCC 14028) and are significantly influenced by host cell types (e.g., epithelial cells vs. macrophages) and their physiological states. The research findings discussed in this section are mainly derived from established infection models and should therefore be interpreted within these specific contexts. Metabolic reprogramming is the core bridge connecting “intracellular survival” and “immune evasion”. During the intracellular parasitic process, *S*. Typhimurium actively regulates the metabolic flux of host cells to create favorable conditions for its own proliferation and immune escape (**Figure 4**).

#### 6.2.1 Hijacking host glycolysis for replication

The bacterium utilizes its T3SS effector protein SopE2 to activate the host’s Cdc42, thereby stimulating the PI3K-Akt signaling pathway. Activation of this signaling pathway upregulates the expression of glycolysis-related enzymes, prompting a shift in the energy metabolism of macrophages from oxidative phosphorylation (OXPHOS) to glycolysis, resulting in the accumulation of glycerol-3-phosphate (G3P) in the cytoplasm. Subsequently, *S*. Typhimurium uses its own glycerol-3-phosphate dehydrogenase (G3PDH) to reintroduce host-derived G3P into its tricarboxylic acid (TCA) cycle, thereby significantly enhancing the replication efficiency and quantity of the bacterium within macrophages under energy-sufficient conditions (Jiang et al., 2021).

#### 6.2.2 Metabolic adaptation to virulence-inducing conditions

Under relevant virulence-inducing conditions (such as LPM medium with low pH, low magnesium, and low iron), *S*. Typhimurium can switch its carbon source utilization from glycerol catabolism to glucose. Amino acids accumulate intracellularly, but the activity of their synthetic pathways decreases. Meanwhile, virulence-related pathways such as lipopolysaccharide (LPS) biosynthesis and fatty acid metabolism are preferentially enhanced to adapt to the nutrient-limited environment inside the host (Kim et al., 2013). This bacterial-driven metabolic change enhances the host’s glycolytic activity, leading to a large accumulation of reduced nicotinamide adenine dinucleotide (NADH) and a decrease in the NAD^+^/NADH ratio. The change in this ratio specifically promotes the secretion of pro-inflammatory factors while inhibiting the normal response of type I interferons, thereby creating an energy-rich and immune-suppressed microenvironment for the intracellular survival of *S*. Typhimurium (Buckner et al., 2011).

#### 6.2.3 Intrinsic metabolic regulation of bacteria

To maintain its own redox homeostasis, *S*. Typhimurium also regulates its NAD metabolism. Key genes such as *nadA* and *pmtC* are globally regulated by *nadR* and are induced to express under anaerobic conditions to ensure NAD synthesis and the utilization of exogenous precursors, thereby enhancing its adaptability in the host intestine (Klabunde et al., 2023). The transcription factor SlyA not only regulates virulence but also directly affects glucose metabolism and lipid metabolism. The deletion of *slyA* leads to a decrease in the levels of pyruvate, ATP precursors, and TCA cycle intermediates, while the expression of genes related to malate synthase and ATP synthesis is also downregulated. In terms of lipid metabolism, the deletion of *slyA* causes abnormal metabolism of various fatty acids and phospholipids, and downregulates multiple lipid synthesis-related genes (Tian et al., 2021).

#### 6.2.4 Response to environmental stress and disturbance

In response to environmental stress, the metabolic adaptability of *S*. Typhimurium may be disrupted by external physical factors. For example, ultrasonic waves induce metabolic disorders inside bacteria (including impaired energy metabolism, oxidative stress, and lipid metabolism imbalance) by physically damaging cell structures and membrane permeability, thereby synergistically enhancing the bactericidal effect of chlorine dioxide (Luo et al., 2022). In an anaerobic environment, metabolic reprogramming is regulated by FNR. Comparative proteomic analysis shows that FNR enables bacteria to adapt to the hypoxic intestinal environment by down-regulating proteins related to the TCA cycle enzymes, aerobic electron transport, and oxidative phosphorylation, while up-regulating proteins related to pyruvate metabolism, glycerol metabolism, and glycerophospholipid metabolism. In addition, FNR negatively regulates the utilization of ethanolamine, positively regulates flagellar synthesis, motility, and chemotaxis, and indirectly affects bacterial virulence by activating Fis (Behera et al., 2020).

#### 6.2.5 Association between metabolism, virulence, and stress response

These metabolic regulatory mechanisms are closely related to the expression of bacterial virulence. For example, the effector protein SseJ induces PHB2-dependent mitophagy in host cells, a process that can clear dysfunctional mitochondria, reduce the production of reactive oxygen species (ROS), and provide lipid metabolism precursors for bacteria (Kolodziejek and Miller, 2015; Sun et al., 2025). However, reactive oxygen species may still damage key iron-sulfur cluster-containing enzymes in the bacterial tricarboxylic acid (TCA) cycle, leading to citrate accumulation and metabolic network remodeling; it is noteworthy that direct knockout of tricarboxylic acid cycle enzymes does not directly weaken virulence, whereas the overall accumulation of reactive oxygen species is the key factor affecting virulence (Noster et al., 2019b). To cope with reactive oxygen species stress, *S*. Typhimurium reduces oxidative damage and DNA breaks through cysk-mediated hydrogen sulfide (H_2_S) accumulation, enhancing resistance to fluoroquinolone antibiotics, a mechanism that is particularly important in the macrophage environment rich in reactive oxygen species (Frávega et al., 2016). The effector protein SseK1 can further improve the intracellular survival rate of bacteria by enhancing host glycolysis and inhibiting pro-inflammatory cytokines (Lu et al., 2021).

The SPI-2 effector SteE (also known as SarA) is a typical example of directly reprogramming the phenotype of host immune cells to establish a suitable living environment. SteE is a phosphothreonine lyase that can cleave and inactivate the host kinases GSK3α/β. This action leads to the sustained activation of the transcription factor STAT3, prompting infected macrophages to shift toward an anti-inflammatory M2-like state. This effector-mediated immunometabolic reprogramming inhibits the bactericidal response and creates a microenvironment conducive to the long-term replication and persistent survival of bacteria (Okumura et al., 2022). When the level of reactive oxygen species remains high, the ArcAB two-component system is activated. It drives the production of virulence factors by upregulating the expression of antioxidant genes and cooperating with the SoxRS/OxyR regulatory system, thereby comprehensively enhancing the survival and reproductive capabilities of bacteria in the infectious environment (Pardo-Esté et al., 2019). The Cpx envelope stress system regulates the phosphorylation state of CpxR through the phosphatase activity of CpxA, thereby inhibiting the expression of the SPI-1 key gene *hilA*. In addition, it directly targets various virulence factors and metabolic regulatory genes, linking 1,2-propanediol metabolism to virulence expression to enhance its competitive survival advantage in the inflammatory intestinal environment (Subramaniam et al., 2019). The transcription factor BolA can further enhance bacterial resistance to oxidative stress and virulence-related metabolic adaptability by regulating the accumulation of metabolites such as acetic acid, valine, alanine, nicotinamide adenine dinucleotide (NAD^+^), succinic acid, glutathione, and putrescine (Graça-Lopes et al., 2019). The m¹G modification of tRNA affects codon decoding efficiency and polypeptide chain elongation rate in a tRNA-dependent manner, leading to a decrease in carbon source utilization efficiency and changes in sensitivity to amino acid analogs, which may influence the intracellular adaptability of bacteria by regulating the synthesis of virulence proteins (Fasciano and Hallenbeck, 1992).

#### 6.2.6 Metabolic strategies of the host

The host combats bacterial infections through various metabolic mechanisms. For example, during *S*. Typhimurium infection, host cells generate itaconic acid in mitochondria by activating the transcription factor EB (TFEB) and transport it to SCVs via the Irg1-Rab32-BLOC3 pathway. The accumulation of itaconic acid in SCVs can effectively inhibit bacterial proliferation (Schuster et al., 2022). In infection models, such as the cecum of Wenchang chickens, *Salmonella* infection leads to carbohydrate metabolism disorders, amino acid imbalances, and polyamine accumulation, which reflects the complex metabolic interactions between the host and the pathogen, as well as significant disruptions in the gut microbiota-metabolism axis (Chen et al., 2024). Additionally, host macrophages regulate immune responses by activating glycolysis and altering the NAD^+^/NADH ratio, while *Salmonella* can interfere with this process using effector proteins such as SseK1, fully revealing the core role of metabolic reprogramming in infection pathology.

## 7 Immune escape

### 7.1 Bidirectional regulatory strategy of autophagy pathway

**Figure 5:**
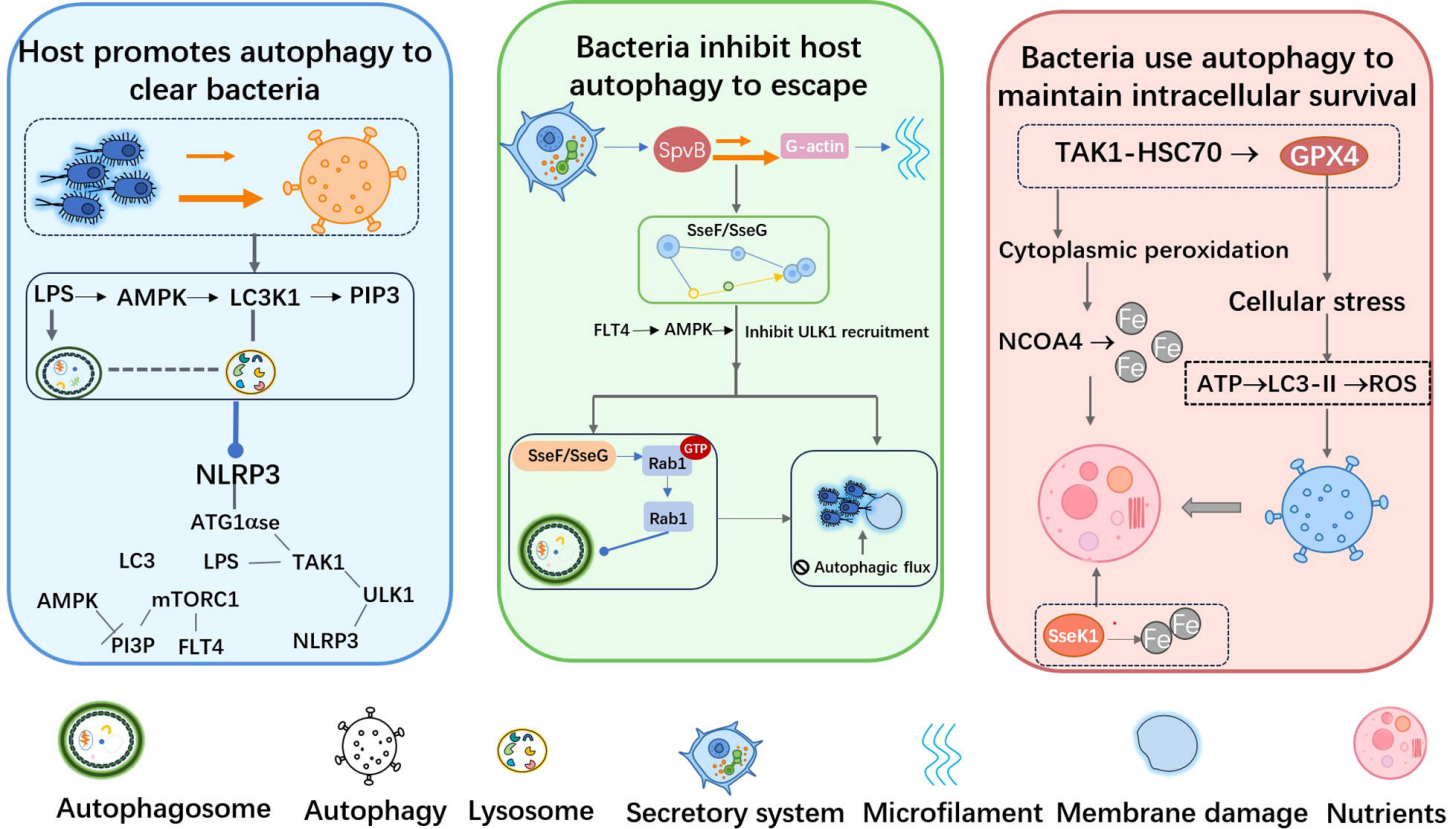
Bidirectional regulation diagram of the autophagy pathway of *S*. Typhimurium. Blue background: It reflects the host’s defense mechanism, that is, initiating autophagy to eliminate the invading *S*. Typhimurium, which is a manifestation of the host’s active resistance to bacteria. Green background: It shows the countermeasures taken by *S*. Typhimurium to avoid being eliminated by autophagy, reflecting its survival adaptability. Pink background: It reveals that *S*. Typhimurium does not simply oppose autophagy, but selectively uses the autophagy pathway to create conditions for its own survival.

It should be noted that the specific mechanisms and outcomes of autophagy regulation by *S*. Typhimurium may vary across different bacterial strains and host cell types. The following description is based largely on established experimental models and may not fully represent all infection contexts. During the long-term co-evolution process between hosts and pathogens, autophagy, as a conserved cellular self-degradation mechanism, has become a key link in anti-infectious immunity. When *S*. Typhimurium breaks through the host cell membrane barrier, the cell will immediately activate a multi-faceted autophagy defense network (**Figure 5**, blue panel).

#### 7.1.1 Host autophagy initiation mechanism

**Figure 6:**
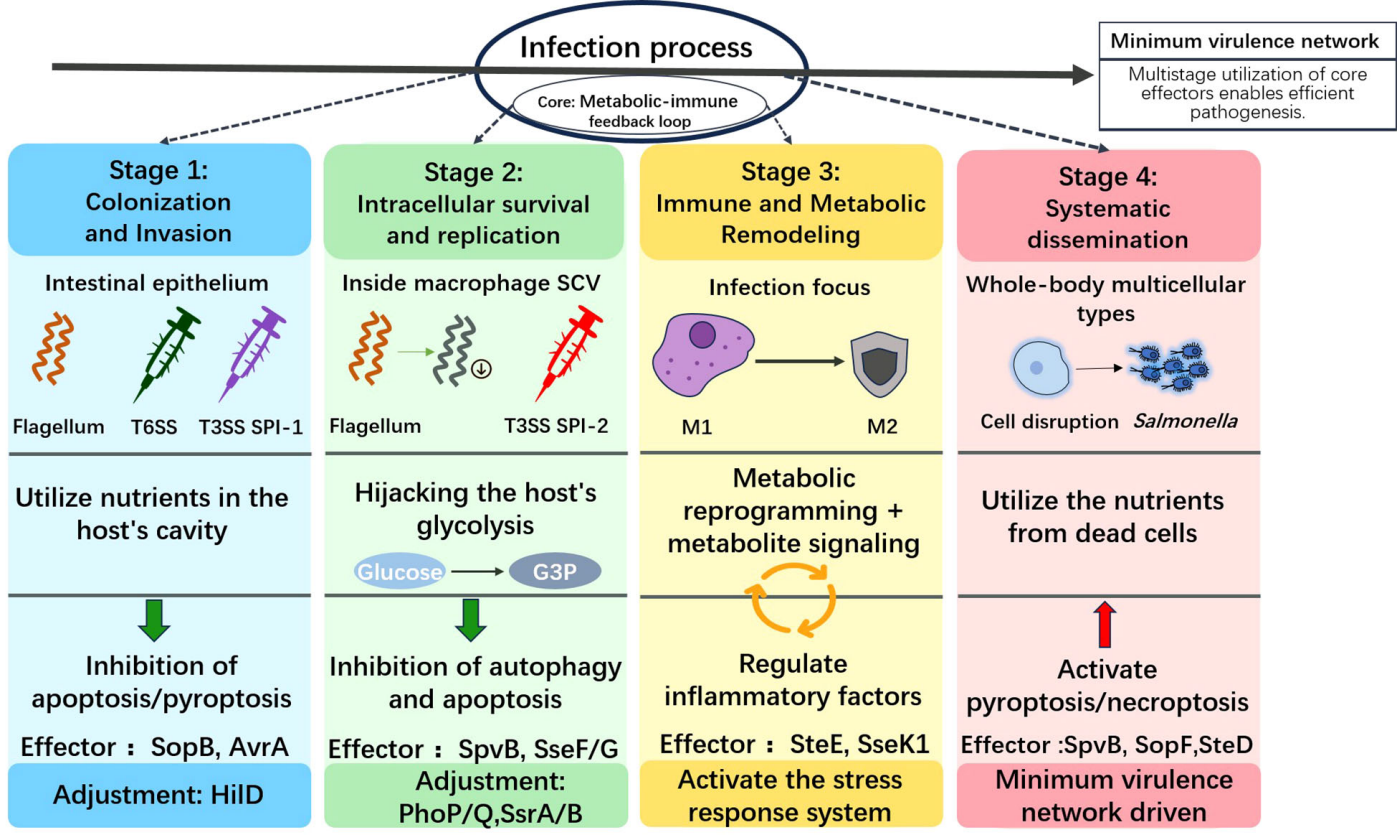
Stage-specific virulence program of *S*. Typhimurium. This model illustrates the coordinated infection cycle: initial invasion via flagella, T6SS and SPI-1 T3SS establishes the SCV while suppressing cell death. Within the SCV, SPI-2 effectors stabilize the niche and hijack host glycolysis for replication. Immunometabolic reprogramming by effectors like SopE2 and SteE then creates a pro-survival, anti-inflammatory state. Finally, late-stage effectors (e.g., SpvB) trigger inflammatory cell death to disseminate bacteria and evade adaptive immunity. The entire process is driven by a precise, minimal virulence network.

The initiation of antibacterial autophagy (xenophagy) relies on several key sensing pathways (**Figure 6**, blue panel). V-ATPase is an inner membrane homeostasis sensor. When sensing lysosomal stress, its V1H subunit undergoes a conformational change. This change enables it to directly recruit the key autophagy protein ATG16L1, drive LC3 lipidation, and initiate the non-canonical autophagy pathway (Xu et al., 2019). Meanwhile, lipopolysaccharide (LPS) on the bacterial surface is recognized by host pattern recognition receptors, activating transforming growth factor-β-activated kinase 1 (TAK1). TAK1 promotes the production of phosphatidylinositol 3-phosphate (PI3P) through the AMPK-mTORC1-ULK1 signaling axis, providing the necessary membrane platform for autophagosome formation (Liu et al., 2018). In addition, the receptor tyrosine kinase FLT4 (VEGFR3) plays a unique role. Upon membrane damage, FLT4 promotes the binding of LC3-II to the membrane, enhances the fusion of autophagosomes with lysosomes by activating AMPK, and crucially, it can inhibit the excessive activation of the NLRP3 inflammasome to balance pyroptosis and autophagy, preventing harmful inflammation while clearing bacteria (Ma et al., 2022).

#### 7.1.2 Bacterial effector proteins that disrupt autophagy

*S*. Typhimurium has evolved sophisticated strategies to disrupt this host defense (**Figure 6**, green panel). The bacterium injects effector proteins through T3SS, which specifically target different stages of autophagy. The ADP-ribosyltransferase SpvB (introduced in section 4) inhibits actin polymerization by modifying G-actin. This action disrupts the localization of the VPS34-Beclin1-PI3K-III complex on phagosomes, interrupts autophagic flux, and enhances inflammatory signals (Chu et al., 2016). In addition, the effector proteins SseF and SseG function as GTPase-activating protein (GAP) mimics for Rab1A. By locking Rab1A in its GDP-bound state, they inhibit the interaction of Rab1A with the TRAPPIII guanine nucleotide exchange factor complex. This prevents the recruitment of ULK1 kinase to the initiation site, blocks the synthesis of PI3P, and effectively inhibits autophagosome maturation (Feng et al., 2018).

#### 7.1.3 Selective utilization of host autophagy components

In some cases, *S*. Typhimurium may not only inhibit autophagy but also selectively utilize its components for its own benefit (**Figure 6**, pink panel). For example, by regulating specific autophagy-related processes, the bacterium may potentially obtain nutrients or alleviate excessive cellular stress, which would otherwise damage its living environment. This flexible bidirectional regulatory strategy, encompassing the inhibition, disruption, and even selective utilization of autophagy, reflects *S*. Typhimurium ‘s profound intervention and remodeling of the host’s core defense mechanisms, transforming a powerful host defense into a controllable and sometimes even beneficial aspect of its intracellular lifestyle.

### 7.2 Synergistic regulation of apoptosis and pyroptosis

The manipulation of host cell death pathways by *S*. Typhimurium exhibits considerable complexity and context-dependency, which may be influenced by bacterial strain-specific effector repertoires and the physiological state of the host cell type encountered. The following summary outlines general paradigms observed in common laboratory models. *S*. Typhimurium, through its T3SS effector protein network, spatiotemporally and specifically coordinates the regulation of host cell apoptosis, pyroptosis, and necroptosis, forming a functionally redundant and mutually synergistic cell death regulatory system aimed at evading immune clearance and promoting transmission.

This regulation is strategically tailored to distinct tissue niches encountered during infection. In the intestinal epithelial niche during early invasion, effector proteins such as SopB and AvrA maintain barrier integrity by inhibiting apoptosis and pyroptosis, thereby facilitating the establishment of a local infection focus. Within the phagosomal niche of professional phagocytes like macrophages, the bacteria prioritize inhibiting autophagy and apoptosis to stabilize the SCVs, securing a replicative compartment and prolonging host cell survival. As the infection progresses to a systemic dissemination phase, the strategy shifts across various cell types toward activating inflammatory death modalities such as pyroptosis or necroptosis via effectors like SpvB and SopF. This promotes the release of bacteria from exhausted host cells, and the ensuing inflammation disrupts tissue architecture and recruits new susceptible cells, thereby driving systemic spread. Therefore, the regulation of cell death is a dynamic adaptation strategy of bacteria to meet the needs of their ecological niches, with its core purpose always centered around the phased survival goals of bacteria: achieving stable colonization at protective sites or efficiently spreading to new hosts (**Table 1**).

**Table 1:** Stage-specific manipulation of host cell death by *S*. Typhimurium.

Infection stage/Niche	Dominant effectors	Targeted death pathway	Outcome for bacterium
Early intestinal invasion (Epithelial cells)	SopB, AvrA	Inhibits Apoptosis & Pyroptosis	Maintains epithelial barrier integrity, establishes initial infection focus.
SCV maturation & intracellular replication (Macrophages)	SpvB, SseF/G, SseJ	Inhibits Autophagy; Modulates Apoptosis	Prevents lysosomal degradation, secures replicative niche, prolongs host cell survival.
Late intracellular/Systemic dissemination (Multiple cell types)	SpvB, SopF, SseK1/3	Promotes Necroptosis/PANoptosis; Fine-tunes Pyroptosis	Facilitates escape from spent host cells, induces pro-inflammatory response to damage barriers and recruit new targets.

#### 7.2.1 Regulation of apoptotic pathways

This bacterium utilizes a series of effector proteins to precisely control apoptosis, primarily to prolong the survival time of host cells during the establishment of infection. SopB plays a central role in inhibiting apoptosis by activating the Akt survival pathway (The molecule starts with the regulation of the SCV membrane, see Section 6.1 for details), which ultimately blocks the intrinsic apoptotic pathway (García-Gil et al., 2018; Guo et al., 2021). AvrA downregulates the expression of pro-apoptotic and inflammatory genes by acetylating and inhibiting key components of the NF-κB and MAPK pathways (Jiao et al., 2020). In contrast, SopD can activate caspase-3/7 in intestinal epithelial cells, inducing apoptosis to disrupt the mucosal barrier and enhance local inflammation; this seems contradictory but may promote bacterial invasion (Lian et al., 2021). The effector protein SseJ (See Section 6.2.5 for details) indirectly inhibits the release of apoptotic signals from damaged mitochondria (Li et al., 2025). Additionally, the ADP-ribosylation of actin by SpvB (As described in Section 4) disrupts the cytoskeleton, inhibiting actin-dependent apoptotic signals in the early stage, while in the later stage, it can instead promote necroptosis to aid in dissemination (Dong et al., 2022). E3 ubiquitin ligases such as SspH1/2 add another layer of regulation by modulating the activity of caspase-8 and RIPK1 (key nodes in apoptotic and necroptotic signaling) (Delyea et al., 2024).

#### 7.2.2 Activation and inhibition of pyroptosis

The regulation of pyroptosis revolves around the assembly of inflammasomes. *S*. Typhimurium can both trigger and inhibit this inflammatory cell death. The guanine nucleotide exchange factors SopE and SopE2 activate Rac1/Cdc42, promote the assembly of the NAIP/NLRC4 inflammasome, and trigger caspase-1-dependent pyroptosis (Friebel et al., 2001; Kelley et al., 2019). Bacterial flagellin (FliC) can also activate NAIP/NLRC4, and by targeting mitochondrial VDAC, it can simultaneously induce pyroptosis and inhibit apoptosis (Sukumaran et al., 2010). Host-derived reactive oxygen species (ROS) and cathepsins further promote the activation of the classical NLRP3 inflammasome (Gram et al., 2021).

To prevent excessive pyroptosis from destroying its replicative niche, the bacterium deploys inhibitory effector proteins. SopF inhibits caspase-8 through the PDK1-RSK pathway, reducing pyroptosis and apoptosis while favoring necroptosis (Yuan et al., 2023). SseK1 and SseK3 (see section 4) block TNFα signaling through arginine GlcNAcylation, inhibiting TNF-induced cell death pathways (Günster et al., 2017). SopB directly inhibits the assembly of the NLRP3 inflammasome (García-Gil et al., 2018), SseL deubiquitinates NLRP3 and ASC to reduce their aggregation, and SpvC dephosphorylates MAPK to attenuate inflammatory signaling (Wu et al., 2018).

#### 7.2.3 Comprehensive control of PANoptosis levels

This regulation extends to a comprehensive cell death platform known as PANoptosis. SopF plays a key role in this process; it inhibits caspase-8 while activating MLKL, thereby affecting the crosstalk between apoptosis, pyroptosis, and necroptosis. When intracellular bacterial replication reaches a high level, complexes such as Casp8-RIPK1-PANoptosome are formed, leading to the synergistic activation of multiple death pathways (Barth and Aktories, 2011). The co-activation of caspase-11 (non-classical) and caspase-1 (classical) further amplifies the inflammatory pyroptotic response, influencing the overall outcome of the infection.

#### 7.2.4 Supplementary mechanisms coordinating host cell death

In addition to the apoptotic and pyroptotic pathways, *S*. Typhimurium also coordinately regulates the host cell death process through other mechanisms. Among them, the PhoP–PhoQ two-component system modifies the structure of bacterial LPS, reducing its ability to be recognized by the host NLRP3 inflammasome, and participates in regulating the expression timing of SPI-1 and SPI-2 effector proteins, thereby optimizing the infection strategy in terms of time and space. The autophagy related protein p62 (SQSTM1) is degraded in the early stage of infection but reaccumulates 4–6 hours after infection; this dynamic change, in synergy with the N-acetylglucosamination modification mediated by the SseK1/3 effector proteins, jointly promotes the degradation of ASC, a key component of the inflammasome, forming a two-layer inhibitory mechanism on inflammasome signaling (Hu et al., 2017). At the regulatory level of Rho GTPase, SopB, SopE, and SopE2 promote cytoskeletal rearrangement by activating Rho family proteins, while SpvB inhibits their activity through ADP-ribosylation. These two functionally form a spatiotemporal conflict, dynamically regulating the cytoskeleton and death signals (Barth and Aktories, 2011). Furthermore, SopE can directly target the endoplasmic reticulum autophagy receptor FAM134B, inhibiting its oligomerization, thereby repressing the endoplasmic reticulum autophagy (ER-phagy) process and further impairing the host’s innate immune defense capabilities (Gatica et al., 2025).

## 8 A comprehensive perspective on *Salmonella* virulence and host interactions

This section aims to integrate the aforementioned discussions and elaborate on the pathogenic mechanism of *S*. Typhimurium from a holistic perspective. The key to its success lies in the temporal coordination and functional integration of multiple virulence systems, forming a dynamically responsive network that interacts with the host (**Figure 6**).

### 8.1 From colonization to transmission: activation of virulence

The process of *Salmonella* infecting its host is dominated by regulatory cascades. The core regulatory factor HilD initiates the SPI-1 T3SS-mediated invasion program by sensing host environmental signals. After successful internalization and formation of the SCV, the microenvironment within the vesicle is sensed by systems such as PhoP/Q and SsrA/B, which in turn coordinate the transcriptional program switch from SPI-1 to SPI-2 effectors, marking the transition to the intracellular persistence phase. This switch is not absolute; evidence suggests that HilD can also affect SPI-2, indicating that these regulatory nodes are interconnected. Notably, recent studies using gene minimization approaches have revealed that a minimal network consisting of only a few core effector proteins, combined with additional effectors such as SpvB, is sufficient to drive systemic infection and intercellular spread of *Salmonella* in tissues like the spleen (Burford et al., 2025). These studies defined a core “θ network” that supports intracellular vacuolar replication, which must synergize with the spv operon (particularly SpvB) to overcome host immune bottlenecks and establish lethal systemic disease (Chen et al., 2021).

In addition to T3SS, other virulence systems are also integrated into this network in a temporal sequence. The T6SS, regulated by factors such as Fur, mainly functions in the initial colonization stage to compete with the microbiota. The flagellar system is crucial for early motility and adhesion; however, in host cells, it is often downregulated in response to acidic pH through regulatory factors like CadC/YdiV, which represents a strategy to reduce immunogenicity. Furthermore, the core regulatory factor of the flagellar system, the FlhD_4_C_2_ complex, and SPI-1 genes jointly receive signal inputs from global regulatory factors such as FNR, thereby ensuring their coordinated role in the anaerobic environment of the intestine.

### 8.2 The feedback loop among metabolism, virulence, and immunity

**Figure 7:**
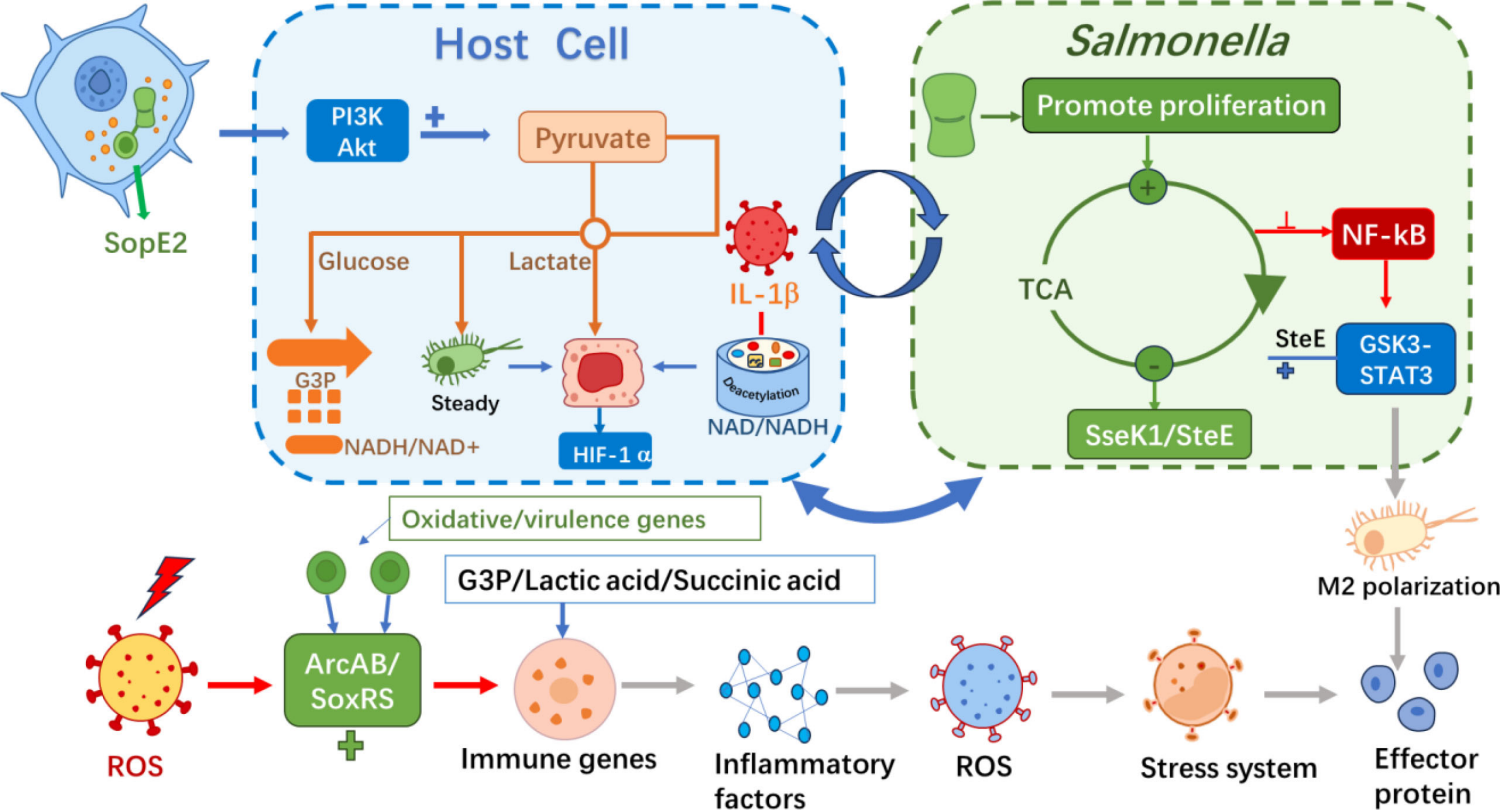
The core metabolic-immune feedback loop. This conceptual diagram depicts how bacterial-driven host metabolic reprogramming initiates a continuous dialogue with immunity. Effectors like SopE2 induce glycolysis, providing nutrients (e.g., G3P) and generating signaling metabolites (e.g., succinate, lactate). These metabolites influence host inflammatory responses and epigenetic states. The resulting metabolic stress (e.g., ROS) feeds back to activate bacterial virulence and stress regulators (e.g., SoxRS, ArcAB). Simultaneously, effectors like SseK1 and SteE directly blunt host immune signaling. This creates a self-reinforcing cycle that favors bacterial persistence.

Metabolic reprogramming is a core pillar of *S*. Typhimurium ‘s strategy, serving both as a means of nutrient acquisition and a powerful mechanism for immune regulation, forming a self-reinforcing feedback loop (**Figure 7**). Bacteria redirect host cell metabolism toward glycolysis through effector molecules such as SopE2, which not only provides a carbon source for bacterial replication but also alters the host metabolome. These metabolic changes themselves act as signaling molecules; for example, succinate can stabilize HIF-1α to promote inflammation.

Crucially, this metabolically altered state feeds back to influence bacterial virulence and host defense. Oxidative stress generated by metabolic changes activates bacterial stress response systems, which in turn upregulate antioxidant defenses and more virulence factors. Meanwhile, effectors such as SseK1 and SteE directly target immune signaling hubs, inhibiting antimicrobial responses through post-translational modifications. Thus, metabolism, bacterial virulence expression, and host immunity are in constant dialogue, with the pathogen attempting to steer the dialogue toward a nutrient-rich and immunosuppressive tolerant state. Single-cell analyses further reveal that the synergistic action of a minimal network of effectors is crucial for bacteria to overcome immune bottlenecks imposed by MyD88 and IFN-γ signaling pathways in tissues such as the spleen (Burford et al., 2025).

### 8.3 Regulation of the cell death network strategic

The interaction between *S*. Typhimurium and host cell death pathways is highly strategic and environment-dependent. The choice to inhibit or induce specific death pathways depends on the stage of infection and the ecological niche. In the early stages of epithelial cell infection, inhibiting apoptosis and pyroptosis can maintain the integrity of the epithelial barrier and promote stable colonization. Within the macrophage SCV, inhibiting autophagy is crucial for preventing lysosomal degradation and ensuring a replicative niche. Minimal network studies have confirmed that the ADP-ribosyltransferase activity of SpvB plays a key role in inhibiting the cytoskeleton and promoting bacterial dissemination from specific monocyte populations, thereby breaking through the bottleneck of tissue infection (Chen et al., 2021).

However, the relationship with cell death is not purely antagonistic. At later stages or under stress, bacteria can trigger pro-inflammatory death modes to facilitate escape and dissemination, while the inflammatory response can disrupt tissues and recruit more target cells. Effector molecules exert precise control at key nodes regulating the crosstalk of different death pathways, enabling bacteria to fine-tune the outcome. Therefore, the manipulation of cell death is a dynamic strategy that balances the need for intracellular persistence and eventual systemic dissemination.

In conclusion, the successful pathogenesis of *S*. Typhimurium stems from a spatiotemporally coordinated network in which secretion systems, metabolic hijacking, and immune evasion are closely linked. Emerging technologies such as gene minimization and single-cell analysis have shown that a small network consisting of core effectors and auxiliary factors is sufficient to drive complex infection processes (Chen et al., 2021; Burford et al., 2025).

## 9 Conclusions and future prospects

The mechanism by which *S*. Typhimurium parasitizes host cells is essentially a survival program encoded in its genome. This program is highly integrated and can respond to environmental changes. It activates virulence systems such as SPI-1, SPI-2, T6SS, and flagella in stages, actively seizes the host’s metabolic network, and strategically regulates the death process of host cells, ultimately achieving good adaptation to and manipulation of the host environment. The core of this mechanism lies in a streamlined and efficient virulence network, which uses the minimum genetic components to achieve the best survival effect.

In this network, core regulatory factors such as HiID can sense stress signals within the host cell. For example, signals like a low-magnesium environment and acidic pH value are converted into activation instructions for virulence devices. The SPI-1, SPI-2 T3SS, T6SS, and flagellar system are functionally complementary and work together. They play important roles in three key links: bacterial invasion of the host, intracellular colonization and survival, and competition for living space, respectively. It is worth noting that bacteria can not only seize nutrients from the host to meet their own reproduction through metabolic reprogramming but also change the surrounding immune environment. At the same time, bacteria can also perform bidirectional and precise regulation on cell death processes such as autophagy, apoptosis, and pyroptosis. At the appropriate time, on the one hand, they maintain the stability of host cells to ensure their own survival; on the other hand, they trigger the spread of inflammation to expand the infection range, ultimately successfully establishing and maintaining the infection focus.

In the future, figuring out the changes in this complex interaction network across different times and spaces will be both a major challenge and an important opportunity. Emerging single-cell and spatial multi-omics technologies will reveal the respective differences between the host and the pathogen during the infection process. Models like organoids, which are close to the physiological state of the human body, can accurately reconstruct the maturation process of SCVs and the metabolic interactions between the host and the pathogen. Combining proteomics and metabolomics to systematically identify the targets of effector proteins in the host can more precisely pinpoint the key molecules in the pathogenesis. Importantly, advancements in these basic research areas are driving the rational design of new intervention methods. These methods either disrupt the virulence network of the pathogen or enhance the host’s defense capabilities, a strategy that may avoid the drug resistance issues caused by traditional antibiotics.

The most promising application directions include the development of small-molecule or nucleic acid-based anti-virulence drugs targeting core regulatory factors such as HiID and SsrB. Such drugs can attenuate the pathogenic process of bacteria from the source of transcription. Other auxiliary methods involve using precise inhibitors to directly render bacteria non-pathogenic. These inhibitors can block the conserved needle complex of the T3SS or the catalytic domains of key effector proteins such as AvrA and SpvB, thereby preventing bacterial invasion and the delivery of effector proteins. Host-directed therapy is another strategy. For example, restricting bacteria’s access to essential nutrients such as NAD^+^ and β-alanine, inhibiting pathogen-specific metabolic enzymes like G3PDH to induce metabolic starvation in bacteria, or increasing the host’s own antimicrobial metabolites such as itaconic acid. Immunomodulatory strategies that can regulate the balance of cell death at the infection site or adjust the local immune metabolic state also have therapeutic potential. Additionally, next-generation subunit vaccines contain conserved components of the secretion system or neutralizing epitopes of key effector proteins. Such vaccines can induce immune responses specifically blocking virulence functions, providing a mechanism-based approach for achieving long-term protection.

In conclusion, systematically studying the pathogenic mechanism of *S*. Typhimurium not only clarifies its complex survival patterns but also provides various precise solutions to address the increasingly severe problem of antibiotic resistance. To translate these strategies, such as anti-virulence drugs and secretion system inhibitors, into host-targeted metabolic therapies and mechanism-based vaccines, long-term interdisciplinary collaboration across multiple fields including microbiology, immunology, structural biology, and medicinal chemistry is required.
